# An Adaptive Hybrid Correlation Kriging Approach for Uncertainty Dynamic Optimization of Spherical-Conical Shell Structure

**DOI:** 10.3390/ma18153588

**Published:** 2025-07-30

**Authors:** Tianchen Huang, Qingshan Wang, Rui Zhong, Tao Liu

**Affiliations:** 1 College of Mechanical and Electrical Engineering, Central South University, Changsha 410083, Chinaliutaosr@csu.edu.cn (T.L.); 2 State Key Laboratory of Precision Manufacturing for Extreme Service Performance, Central South University, Changsha 410083, China

**Keywords:** laminated spherical-conical shell, uncertainty optimization, adaptive hybrid correlation kriging, improved multi-objective salp swarm algorithm

## Abstract

In this paper, an uncertainty optimization method based on the adaptive hybrid correlation Kriging surrogate model is proposed to optimize the ply angles of laminated spherical-conical shells. First, equations of motion of laminated spherical-conical shells are constructed to calculate the vibration characteristics. Then, this paper proposes a Kriging surrogate model with adaptive weight hybrid correlation functions and validates its accuracy. Based on this framework, the weight distribution of the surrogate model for uncertain parameters in laminated spherical-conical shells under different ply angles is analyzed. To address the uncertainty optimization problem in laminated spherical-conical shell structures, an Improved Multi-objective Salp Swarm Algorithm is developed, and its optimization efficacy is systematically validated. Furthermore, an adaptive hybrid correlation Kriging surrogate model is reconstructed, incorporating both uncertainty parameters and design variables as inputs, with the peak vibration displacement and fundamental frequency serving as the output responses. The uncertainty optimization results confirm that the proposed methodology, along with the enhanced Kriging modeling strategy, exhibits both applicability and computational efficiency for such engineering applications.

## 1. Introduction

Laminated spherical-conical shell structures have been widely utilized in aerospace, marine, and defense engineering fields due to their excellent lightweight properties, high specific strength, and outstanding design adaptability [1,2]. Unlike traditional metallic materials, laminated composites possess a distinct advantage in that their mechanical performance can be precisely tailored through strategic adjustment of lamination schemes [3,4,5]. In practical engineering scenarios, these structures often operate under complex and severe environmental conditions. Improper design of ply angles may lead to structural fatigue failure under dynamic loads. Consequently, the optimal design of ply angles to minimize vibration response represents a critical research focus for enhancing the performance of laminated spherical-conical shells.

Recent advancements in computational optimization methodologies have demonstrated remarkable potential in addressing complex structural optimization challenges. Hybrid optimization models integrating neural networks with swarm intelligence techniques, along with enhanced algorithms incorporating machine learning approaches, have exhibited outstanding performance improvements in engineering optimization problems [6,7,8]. These state-of-the-art intelligent optimization techniques have shown particular efficacy in handling multi-objective optimization problems, especially for engineering applications involving conflicting performance criteria such as simultaneous frequency and displacement response optimization [9,10]. Contemporary research in composite materials has further substantiated the effectiveness of advanced optimization frameworks in solving various engineering problems, including damage identification and performance prediction applications [11]. These research achievements provide crucial theoretical foundations for the current study.

Current optimization research predominantly considers deterministic parameters, as evidenced by contributions from Wang et al. [12], Cheng et al. [13], Dat et al. [14], and Duc et al. [15]. While deterministic optimization methods excel in solving problems with well-defined mathematical models, their solutions may deviate from practical requirements when systems involve uncertain factors such as parameter fluctuations or environmental disturbances. Consequently, uncertainty-based optimization approaches (e.g., robust optimization and stochastic programming) have emerged as research hotspots, aiming to enhance solution reliability through quantitative uncertainty analysis. In engineering practice, accurately quantifying uncertain parameters in shell structures remains particularly challenging, necessitating mathematical characterization methods [16]. Current uncertainty quantification approaches primarily include probabilistic and non-probabilistic methods. Probabilistic methods provide detailed statistical descriptions but often prove impractical for engineering applications where obtaining precise probability distributions is difficult [17]. In contrast, non-probabilistic methods only require parameter ranges or intervals for analysis, enabling effective uncertainty quantification with minimal information while offering superior flexibility and applicability [18]. This study employs non-probabilistic analysis, which can be further classified into convex analysis and interval analysis methods. Convex analysis typically considers only single-boundary cases of uncertain objective functions and constraints, whereas interval analysis accounts for both boundaries, delivering more comprehensive and accurate results. The theoretical foundation of interval numbers traces back to the 1930s [19], with subsequent developments by Sengupta and Pal [20] significantly advancing interval linear programming and propelling widespread adoption of interval-based uncertainty optimization. This research employs the interval analysis approach to address material uncertainties in laminated spherical-conical shell structures.

The interval analysis method necessitates substantial computational effort due to its intricate dual-layer nested structure, which consequently leads to an inefficient and time-consuming design optimization procedure. Under this context, surrogate model-based optimization design has gradually gained researchers’ attention. Among these models, the Kriging surrogate model—a Best Linear Unbiased Prediction (BLUP) method for Gaussian process regression—demonstrates strong fitting capability and robustness [21]. Due to its excellent approximation ability for nonlinear functions and unique error estimation feature, the Kriging surrogate model has attracted increasing interest and has become one of the most representative and promising surrogate modeling approaches [22,23]. The core of the Kriging model lies in its use of correlation functions to describe spatial dependencies among sample points, thereby predicting responses at unknown locations while quantifying prediction uncertainty. The choice of correlation function directly affects the model’s fitting accuracy and generalization capability [24,25]. Different correlation functions exhibit varying degrees of smoothness and local sensitivity. If the selected correlation function mismatches the spatial variability characteristics of the actual problem, it may lead to overfitting or underfitting, significantly degrading prediction accuracy. Therefore, when constructing a Kriging model, it is crucial to select or design an appropriate correlation function tailored to the problem—or even adopt a hybrid correlation function strategy—to balance global trend approximation and local detail characterization, ensuring both high precision and robustness [26,27,28]. In order to optimize the Kriging model’s prediction performance, the present research proposes an adaptive weighted hybrid correlation function approach to construct a high-fidelity Kriging surrogate model.

Correlation function hybrid techniques have emerged as a novel methodology for enhancing Kriging surrogate model performance. This approach combines complementary correlation functions (e.g., global and local correlation functions) to improve predictive capability [29]. Current integration strategies for Kriging surrogate models can be classified into three categories: global integration (fixed weights), local integration (dynamic weights), and hybrid approaches [30,31,32,33,34]. Existing methods include computationally intensive genetic programming algorithms, and greedy search strategies. While genetic programming suffers from inefficiency due to requiring numerous candidate model constructions, greedy search achieves an automatic correlation function combination through iterative optimization [35,36,37]. Although demonstrating domain-specific advantages, these methods remain computationally prohibitive for sequential optimization scenarios. Notably, simple correlation function combinations do not guarantee performance enhancement and require careful design to prevent overfitting. Consequently, developing hybrid correlation function strategies that simultaneously optimize computational efficiency and modeling accuracy has become a critical research focus in engineering computational experiments.

A comprehensive literature review indicates that while the dynamics behavior of the laminated structure has been extensively investigated, studies focusing on the vibration characteristics of the laminated spherical-conical shells remain notably scarce, particularly regarding displacement uncertainty optimization. Furthermore, although an accurate Kriging surrogate model can substantially enhance computational efficiency, limited attention has been devoted to developing systematic approaches for constructing high-fidelity Kriging models. Bridging this research gap is essential for enabling efficient and robust uncertainty optimization in structural dynamics applications.

This paper proposes an adaptive hybrid correlation function-weighted Kriging surrogate model to accelerate the uncertainty optimization process for the dynamic response of laminated spherical-conical shell structures. Additionally, an enhanced multi-objective optimization algorithm is introduced to improve the coverage of the Pareto front, thereby rendering the optimization results more robust. The structure of this paper is arranged as follows. In Section 2, the motion equations of laminated spherical-conical shells are developed and confirmed. In Section 3, the improvement strategy of the Adaptive Hybrid Correlation Kriging is presented and the performance analysis is conducted. In Section 4, the Improved Multi-objective Salp Swarm Algorithm is introduced, and uncertainty optimization is performed based on the Adaptive Hybrid Correlation Kriging method. Final conclusions are summarized in Section 5.

## 2. Dynamic Model of the Spherical-Conical Shell

### 2.1. Description of the Structure

**Figure 1:**
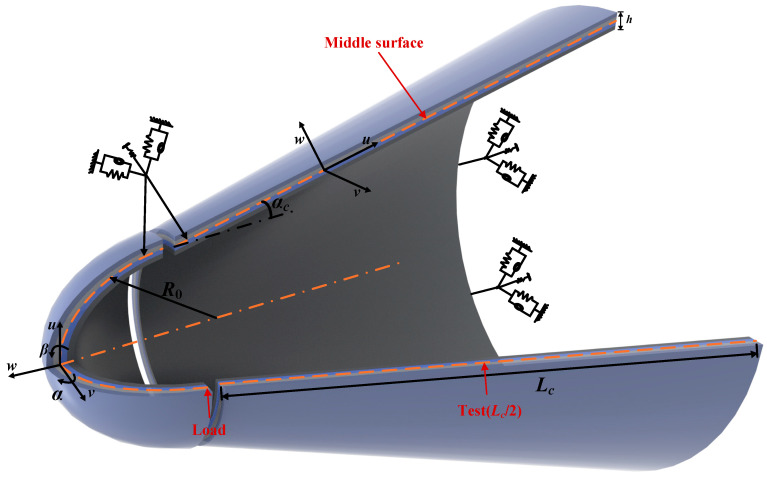
Laminated spherical-conical shell structure.

The laminated spherical-conical shell structure is composed of a combination of spherical and conical configurations, integrating the multidirectional load-bearing capacity of the spherical shell with the axial stiffness of the conical shell. As illustrated in Figure 1, the structure features a single boundary located at the base of the conical shell. In Figure 1, *R*_0_ denotes the radius of the spherical shell, *α_c_* represents the angle between the generatrix of the conical shell and its central axis, *L_c_* indicates the length of the conical shell’s generatrix, and *h* signifies the thickness of the laminated spherical-conical shells. In this study, the load is applied at the junction between the spherical and conical shells, while the response is measured along the same generatrix at a distance of *L_c_*/2 from the loading position.

### 2.2. Equations of Motion for Vibrational Characterization

The laminated spherical-conical shell structure studied in this paper is characterized by a medium-thick shell, so the First-order Shear Deformation Theory (FSDT) is used to construct the equation of motion [38]. Based on the FSDT, the displacement field of laminated spherical-conical shells can be expressed in terms of mid-surface displacements and rotations (*z* = 0) as follows:

(1) $$\begin{array}{l} U(\alpha ,\beta ,z,t)=u(\alpha ,\beta ,t)+z\psi_{\alpha }(\alpha ,\beta ,t)\\ V(\alpha ,\beta ,z,t)=v(\alpha ,\beta ,t)+z\psi_{\beta }(\alpha ,\beta ,t)\\ W(\alpha ,\beta ,z,t)=w(\alpha ,\beta ,t) \end{array}$$

where the rotational displacements *ψ_α_* and *ψ_β_* correspond to the transverse and normal directions (*α* and *β*) of the intermediate surface, with *t* indicating time. The relationship between strain and displacement can be expressed as follows:

(2) $$\begin{array}{ll} \varepsilon_{\alpha }^{0}=\frac{1}{A}\frac{\partial u}{\partial \alpha }+\frac{v}{AB}\frac{\partial A}{\partial \beta }+\frac{w}{R_{\alpha }}, & \varepsilon_{\beta }^{0}=\frac{1}{B}\frac{\partial v}{\partial \beta }+\frac{u}{AB}\frac{\partial B}{\partial \alpha }+\frac{w}{R_{\beta }},\\ \gamma_{\alpha \beta }^{0}=\frac{A}{B}\frac{\partial }{\partial \beta }(\frac{u}{A})+\frac{B}{A}\frac{\partial }{\partial \alpha }(\frac{v}{B}), & k_{\alpha }=\frac{1}{A}\frac{\partial \psi_{\alpha }}{\partial \alpha }+\frac{\psi_{\beta }}{AB}\frac{\partial A}{\partial \beta },\\ k_{\beta }=\frac{1}{B}\frac{\partial \psi_{\beta }}{\partial \beta }+\frac{\psi_{\alpha }}{AB}\frac{\partial B}{\partial \alpha }, & k_{\alpha \beta }=\frac{A}{B}\frac{\partial }{\partial \beta }(\frac{\psi_{\alpha }}{A})+\frac{B}{A}\frac{\partial }{\partial \alpha }(\frac{\psi_{\beta }}{B}),\\ \gamma_{\alpha z}^{0}=\frac{1}{A}\frac{\partial w}{\partial \alpha }-\frac{u}{R_{\alpha }}+\psi_{\alpha }, & \gamma_{\beta z}^{0}=\frac{1}{B}\frac{\partial w}{\partial \beta }-\frac{v}{R_{\beta }}+\psi_{\beta } \end{array}$$

where *A* and *B* are Lamé coefficients. The matrix representation of the constitutive equations governing the stress–strain relationship at the middle surface of the conical-spherical shell is given by the following [39]:

(3) $$\begin{array}{c} \left[\begin{array}{l} Q_{\alpha }\\ Q_{\beta } \end{array}\right]=\lambda \left[\begin{array}{cc} A_{55} & A_{45}\\ A_{45} & A_{44} \end{array}\right]\left[\begin{array}{l} \gamma_{\alpha z}^{0}\\ \gamma_{\beta z}^{0} \end{array}\right] \end{array}$$

where the shear correction factor *λ* is assigned the conventional value of 5/6. The stiffness coefficients *A_ij_* (extensional), *B_ij_* (coupling), and *D_ij_* (bending) are computed according to standard laminate theory, with their detailed formulation available in Ref. [40].

Due to the differences in the geometric models and coordinate systems between spherical shells and conical shells, it is necessary to separately define the parameters in the aforementioned formulas. Specifically, the coordinate systems, Lamé coefficients, and shell curvature radii for spherical shells and conical shells are defined as follows:

(4) $$\begin{array}{l} \mathrm{Spherical}\;\mathrm{shells}:\alpha =\varphi ,\beta =\theta ,A=R_{s},B=R_{s}\sin\varphi ,R_{\alpha }=R_{\beta }=R_{s}\\ \mathrm{Conical}\;\mathrm{shells}:\alpha =s,\beta =\theta ,A=1,B=s\tan\alpha_{0},R_{\alpha }=\infty ,R_{\beta }=s\tan\alpha_{0} \end{array}$$

Building upon the dynamic models of composite spherical shells and conical shells, the coupling relationship between the two structures will be further considered to establish a laminated spherical-conical shells dynamic model. The generalized Lagrange energy function of the laminated spherical-conical shell system is established as follows

(5) $$L=T^{s}+T^{c}-U^{s}-U^{c}-U_{sp}^{s}-U_{sp}^{c}-V_{sc}^{s-c}+W^{F}$$

where *T*, *U*, and *U_sp_* correspond to the kinetic energy, elastic potential energy, and boundary potential energy terms, respectively. The superscript *s* denotes quantities associated with the spherical shell, while *c* represents those corresponding to the conical shell. According to the FSDT, their expressions are given as follows:

(6) $$T=\frac{1}{2}\int_{\alpha_{0}}^{\alpha_{1}}\int_{0}^{2\pi }\left\{\begin{array}{l} I_{0}\left[{\left(\frac{\partial u}{\partial t}\right)}^{2}+{\left(\frac{\partial v}{\partial t}\right)}^{2}+{\left(\frac{\partial w}{\partial t}\right)}^{2}\right]+2I_{1}(\frac{\partial u}{\partial t}\frac{\partial \psi_{\alpha }}{\partial t}+\frac{\partial v}{\partial t}\frac{\partial \psi_{\beta }}{\partial t})\\ +I_{2}\left[{\left(\frac{\partial \psi_{\alpha }}{\partial t}\right)}^{2}+{\left(\frac{\partial \psi_{\beta }}{\partial t}\right)}^{2}\right] \end{array}\right\}ABd\alpha d\beta$$

(7) $$U=\frac{1}{2}\int_{\alpha_{0}}^{\alpha_{1}}\int_{0}^{2\pi }\left(\begin{array}{l} N_{\alpha }\varepsilon_{\alpha }^{0}+N_{\beta }\varepsilon_{\beta }^{0}+N_{\alpha \beta }\gamma_{\alpha \beta }^{0}+M_{\alpha }k_{\alpha }\\ +M_{\beta }k_{\beta }+M_{\alpha \beta }k_{\alpha \beta }+Q_{\alpha }\gamma_{\alpha z}^{0}+Q_{\beta }\gamma_{\beta z}^{0} \end{array}\right)ABd\alpha d\beta$$

(8) $$U_{sp}=\frac{1}{2}\int_{0}^{2\pi }\left\{\begin{array}{l} {\left[k_{u0}u^{2}+k_{v0}v^{2}+k_{w0}w^{2}+k_{\alpha 0}\psi_{\alpha }^{2}+k_{\beta 0}\psi_{\beta }^{2}\right]}_{\alpha =\alpha_{0}}\\ +{\left[k_{u1}u^{2}+k_{v1}v^{2}+k_{w1}w^{2}+k_{\alpha 1}\psi_{\alpha }^{2}+k_{\beta 1}\psi_{\beta }^{2}\right]}_{\alpha =\alpha_{1}} \end{array}\right\}Bd\beta$$

where the coefficients *I_s_* (*s* = 0, 1, 2) represent the mass inertia parameters, as defined in Ref. [41]; *k_u_*_0_, *k_v_*_0_, *k_w_*_0_, *k_α_*_0_, *k_β_*_0_, *k_u_*_1_, *k_v_*_1_, *k_w_*_1_, *k_α_*_1_, and *k_β_*_1_ denote the boundary stiffnesses.

In Equation (5), *V_sc_* represents the coupling spring potential energy at the laminated spherical-conical shell’s structural junction. Based on the continuity conditions of the substructures at the coupling position, a set of elastic couplers comprising linear springs and torsional constraint springs is introduced to satisfy the displacement and physically admissible compatibility conditions at the coupling interface. The expression for *V_sc_* is given as follows:

(9) $$V_{sc}=\frac{1}{2}\iint \left[\begin{array}{l} k_{c}{\left(u^{s}-u^{c}\right)}^{2}+k_{c}{\left(v^{s}-v^{c}\right)}^{2}+k_{c}{\left(w^{s}-w^{c}\right)}^{2}\\ +k_{c}{\left(\psi_{\alpha }^{s}-\psi_{\alpha }^{c}\right)}^{2}+k_{c}{\left(\psi_{\beta }^{s}-\psi_{\beta }^{c}\right)}^{2} \end{array}\right]Rd\alpha dz$$

In Equation (5), *W^F^* represents the work performed by external forces when the shell structure is subjected to external loading, expressed as follows:

(10) $$W^{F}=\int_{\Omega_{f}}\left(f_{\alpha }u+f_{\beta }v+f_{z}w\right)ABd\Omega_{f}$$

where *f_α_*, *f_β_*, and *f_z_* denote the load components acting along the *α*, *β*, and *z* directions of the structure, respectively, and Ω*_f_* represents the load application region on the structure.

The displacement and rotation of the mid-surface are mathematically represented through a series expansion combining Jacobi orthogonal polynomials with Fourier series:

(11) $$u(\alpha ,\beta ,t)=\sum_{m=0}^{M}\sum_{n=0}^{N}P_{m}\left(\alpha \right)\left[\cos\left(n\beta \right){\bar{u}}_{n}^{m}+\sin\left(n\beta \right){\tilde{u}}_{n}^{m}\right]e^{i\omega t}\;v(\alpha ,\beta ,t)=\sum_{m=0}^{M}\sum_{n=0}^{N}P_{m}\left(\alpha \right)\left[\sin\left(n\beta \right){\bar{v}}_{n}^{m}+\cos\left(n\beta \right){\tilde{v}}_{n}^{m}\right]e^{i\omega t}\;w(\alpha ,\beta ,t)=\sum_{m=0}^{M}\sum_{n=0}^{N}P_{m}\left(\alpha \right)\left[\cos\left(n\beta \right){\bar{w}}_{n}^{m}+\sin\left(n\beta \right){\tilde{w}}_{n}^{m}\right]e^{i\omega t}\;\psi_{\alpha }(\alpha ,\beta ,t)=\sum_{m=0}^{M}\sum_{n=0}^{N}P_{m}\left(\alpha \right)\left[\cos\left(n\beta \right){\bar{\psi }}_{\alpha ,n}^{m}+\sin\left(n\beta \right){\tilde{\psi }}_{\beta ,n}^{m}\right]e^{i\omega t}\;\psi_{\beta }(\alpha ,\beta ,t)=\sum_{m=0}^{M}\sum_{n=0}^{N}P_{m}\left(\alpha \right)\left[\sin\left(n\beta \right){\bar{\psi }}_{\beta ,n}^{m}+\cos\left(n\beta \right){\tilde{\psi }}_{\beta ,n}^{m}\right]e^{i\omega t}$$

In this formulation, M denotes the truncation order of the polynomial series. The displacement is expressed using Jacobi polynomials *P_m_*(*α*) of order *m* [42]. The parameter *n* corresponds to the circumferential wave number in the modal vibration pattern, while *N* represents the number of waves considered in the analysis. ${\bar{u}}_{n}^{m}$, ${\tilde{u}}_{n}^{m}$, ${\bar{v}}_{n}^{m}$, ${\tilde{v}}_{n}^{m}$, ${\bar{w}}_{n}^{m}$, ${\tilde{w}}_{n}^{m}$, ${\bar{\psi }}_{\alpha ,n}^{m}$, ${\tilde{\psi }}_{\alpha ,n}^{m}$, ${\bar{\psi }}_{\beta ,n}^{m}$, and ${\tilde{\psi }}_{\beta ,n}^{m}$ denote the generalized coordinate variables.

In substituting Equation (11) into Equation (12), along with the energy generalization function in Equation (5), the equations are subjected to a variational operation using the Rayleigh–Ritz method:

(12) $$\frac{\partial L}{\partial q}=0,q=u_{n}^{m},v_{n}^{m},w_{n}^{m},\psi_{\alpha ,n}^{m},\psi_{\beta ,n}^{m}$$

On the basis of the above, the discretized equation of motion for the laminated spherical-conical shell structure can be obtained:

(13) $$M\ddot{q}+C\dot{q}+Kq=F$$

where **q** represents the global generalized coordinate vector. The system matrices include the generalized mass matrix (**M**), the generalized stiffness matrix (**K**), and a damping matrix (**C**) formulated using Rayleigh’s damping model [43]. The generalized force vector is denoted by **F**.

Assuming harmonic motion, the governing equations of motion for free vibration analysis can be written as follows:

(14) $$\left(K-\omega^{2}M\right)Q=0$$

where *ω* is frequency; **Q** = **q***e^iωt^* denotes the vector of unknown expansion coefficients.

### 2.3. Dynamic Model Verification

In this subsection, the accuracy of the established equation of motion is verified by several numerical examples. The spring stiffness values employed for simulating generalized boundary conditions are summarized in Table 1.

**Figure 2:**
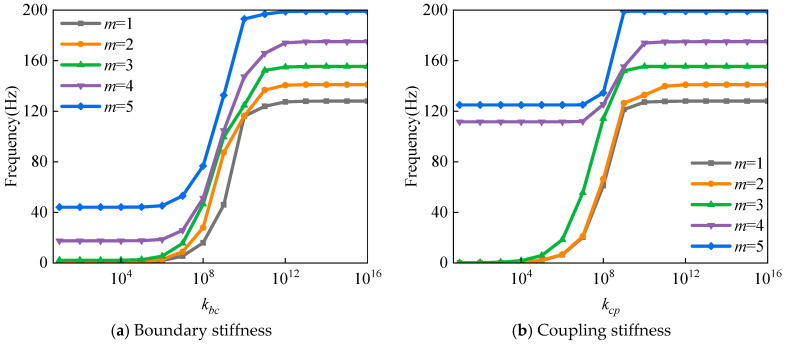
Effect of virtual spring stiffness coefficient on the natural frequency of laminated spherical-conical shell structure.

This study employs virtual springs to simulate the mechanical behavior of connection interfaces, where the stiffness coefficient plays a decisive role in the free vibration characteristics of the structure. Figure 2 systematically presents the influence of virtual spring stiffness on the first five natural frequencies of the structure, with Figure 2a revealing the correlation between boundary virtual spring stiffness *k_bc_* and frequency. The results demonstrate that when the stiffness coefficient *k_bc_* is below 10^5^ N/m, the natural frequencies of the composite shell exhibit low sensitivity to stiffness variations. In the range of 10^5^ N/m to 10^12^ N/m, the natural frequencies show a significant increasing trend with rising stiffness. Beyond 10^12^ N/m, the frequency response stabilizes, indicating that rigid boundary conditions can be effectively simulated at this stage. Figure 2b further presents the influence of virtual spring stiffness *k_cp_* at the spherical-conical shell connection, revealing that the first three frequencies are sensitive to stiffness variations within 10^4^ N/m to 10^12^ N/m, while the fourth and fifth frequencies exhibit noticeable changes only within a narrower range of 10^7^ N/m to 10^10^ N/m. Comprehensive analysis confirms that when the stiffness coefficient exceeds 10^12^ N/m, a stable strong coupling relationship is established between substructures. This conclusion provides important theoretical support for subsequent boundary condition simulations.

Table 2 presents the natural frequencies of the laminated spherical-cylindrical shell structure under different axial displacement function truncation numbers, with comparative results from Ref. [44]. The geometric parameters are defined as follows: *φ*_0_ = 0°, *φ*_1_ = 90°, *α*_0_ = 0, *h* = 0.0022 m, *L_c_* = 0.7 m, and *R*_0_ =0.175 m. The material properties of the homogeneous shell are as follows: *E* = 210 GPa, *ρ* = 7850 kg/m^3^, and *μ* = 0.3. Numerical results demonstrate that the natural frequency characteristics of the laminated spherical-conical shell structure exhibit significant dependence on the truncation number M of the axial displacement function. As the truncation number M increases, the computational results progressively converge toward the reference values from existing literature. When M exceeds 14, the numerical solutions of natural frequencies display satisfactory convergence behavior. Comparative verification with published data confirms that the maximum relative error of the present method’s results remains within 0.1706%, validating both the effectiveness and reliability of the proposed approach in solving free vibration problems of spherical-cylindrical shell structures.

The subsequent analysis will focus on evaluating the performance characteristics of the research subject investigated in this study. The laminated spherical-conical shell structures in this paper use the lamination scheme in which the ply angles are symmetrically laid about the midplane of the structure, set as [*θ*_1_/*θ*_2_/*θ*_2_/*θ*_1_]. The range of values for the ply angle of each layer is the following: −90° ≤ *θ*_1_, *θ*_2_ ≤ 90°. This study utilizes the following material parameters: *E*_1_ = 150 GPa, *E*_2_ = 10 GPa, *μ*_12_ = 0.25, *G*_12_ = *G*_13_ = 6 GPa, *G*_23_ = 5 GPa, and *ρ* = 1450 kg/m^3^. The geometry parameters are as follows: *R*_0_ = 0.5 m, *α_c_* =18°, *L_c_* = 3 m, and *h* = 0.04 m. The thicknesses of each layer are uniformly maintained as identical. Unless otherwise specified, all geometric parameters remain invariant. Table 3 presents the natural frequencies of laminated spherical-conical shell structures under two distinct boundary conditions and different lamination configurations. The validity of the natural frequencies derived from the governing equations is confirmed through comparative analysis with results obtained from finite element simulations, thereby verifying the computational accuracy.

**Figure 3:**
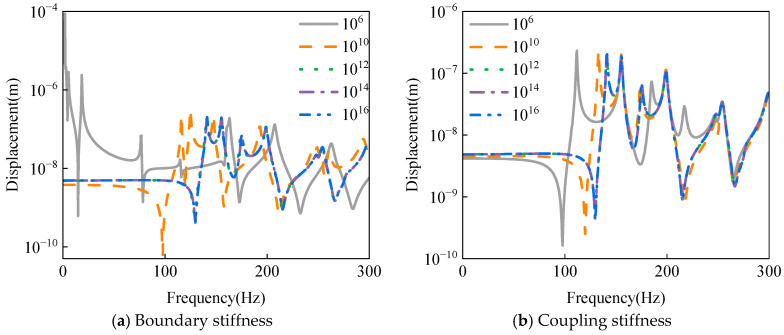
Effect of virtual spring stiffness coefficient on the displacement response of laminated spherical-conical shell structure.

Building upon the aforementioned findings, the virtual spring stiffness coefficient serves as a critical parameter that directly governs the coupling intensity between substructures in the composite shell system. To thoroughly investigate the influence mechanism of spring stiffness on vibration characteristic transmission, this study systematically examines five representative stiffness coefficients: 10^6^ N/m, 10^10^ N/m, 10^12^ N/m, 10^14^ N/m, and 10^16^ N/m. It should be noted that the present study employs a unit point load applied at the spherical-conical junction, with the response extraction point located at *L_c_*/2 along the same generatrix as the loading position. The displacement response analysis is conducted within the 0–300 Hz frequency range. Numerical simulation results presented in Figure 3 demonstrate that when the stiffness coefficient exceeds the critical value of 10^12^ N/m, the structural displacement response stabilizes and ceases to exhibit significant variations with further stiffness increases. This phenomenon confirms the stability characteristics of structural dynamic behavior under strong coupling conditions.

**Figure 4:**
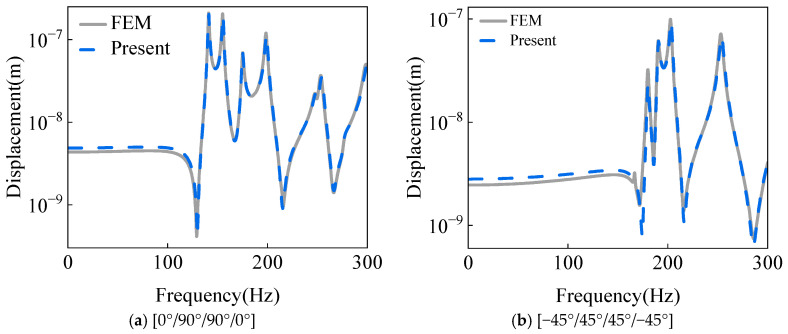
Comparison of the displacement response calculated from the FEM model and the equation of motion.

Figure 4 illustrates the vibration displacement response curves under different lamination schemes, with comparisons provided against finite element simulation results. The spherical-conical shell structure is analyzed under fully clamped (C) boundary constraints. As evidenced by the numerical data in Table 3 and the graphical agreement in Figure 4, the computational outcomes exhibit excellent alignment with reference solutions. This close correspondence validates the accuracy of the derived governing equations for further dynamic analysis.

**Figure 5:**
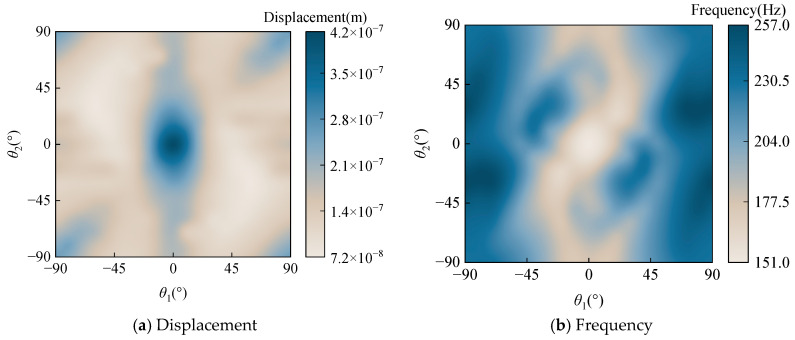
The displacement response and the frequency of the spherical-conical shell structure for different ply angles.

Next in this section, the displacement response and the frequency of the spherical-conical shell structure are investigated for different ply angles. Figure 5a presents the displacements corresponding to different ply angles. As evident from the figure, the structure exhibits maximum displacement when the lamination scheme is [0°/0°/0°/0°]. Figure 5b illustrates the fundamental frequencies for various ply angles, demonstrating that the structure achieves its minimum fundamental frequency under the [0°/0°/0°/0°] lamination schemes. Additionally, both the displacement and fundamental frequency distributions exhibit central symmetry characteristics.

## 3. Adaptive Hybrid Correlation Kriging Analysis

### 3.1. The Introduction to the Correlation Functions

During the following numerical investigation and design optimization, numerous calculations of vibration displacement for the laminated spherical-conical shell are required. Directly solving the established motion equations for each computation proves highly inefficient. To address this issue, establishing a surrogate model becomes essential for enhancing computational efficiency. It is worth mentioning that detailed methodologies for constructing the Kriging surrogate model are available in Ref. [45] and thus will not be redundantly discussed herein. The subsequent section will focus on presenting various types of correlation functions.

The Kriging method originated from the foundational work of Krige [21], with subsequent theoretical advancements made by Matheron [46]. Following its adaptation for Design and Analysis of Computer Experiments (DACE) by Sacks et al. [47], this surrogate modeling technique has gained widespread application in engineering design and optimization. The Kriging model can be mathematically expressed as follows:

(15) $$\hat{y}\left(x\right)=f^{T}\left(x\right)\beta +Z\left(x\right)$$

where $\hat{y}(x)$ denotes the predicted output, **f** corresponds to a vector of basis functions for regression, and *Z* follows a normal distribution with zero mean and covariance matrix Σ, mathematically represented as *Z* ~ *N*(0, Σ):

(16) $$Cov\left[Z\left(x^{\left(i\right)}\right),Z\left(x^{\left(j\right)}\right)\right]=\sigma^{2}Corr\left[Z\left(x^{\left(i\right)}\right),Z\left(x^{\left(j\right)}\right)\right]$$

where *Corr*[*Z*(**x**^(*i*)^),*Z*(**x**^(*j*)^)] denotes the correlation function, which characterizes the dependence between the responses at two sample points **x**^(*i*)^ and **x**^(*j*)^.

At present, commonly used correlation functions include Gaussian, spline, spherical, and Matérn (v = 5/2), with their specific mathematical expressions provided in Table 4 [48]. Specifically, the Gaussian correlation function is particularly suitable for modeling highly smooth, infinitely differentiable functions. While it effectively captures global correlations, it may excessively smooth high-frequency variations. The spline function demonstrates strong adaptability to non-stationary processes and abrupt change regions, making it ideal for modeling steep gradients or discontinuous data, though its performance may become unstable in smooth regions. The Matérn (ν = 5/2) correlation function (hereafter denoted as Matérn5) achieves an optimal balance between smoothness and flexibility. It is well-suited for moderately smooth nonlinear functions, capable of capturing local fluctuations while maintaining continuity. The spherical correlation function exhibits distinct finite-range correlation properties, rendering it particularly effective for low-dimensional problems with pronounced short-range dependence. However, its capacity for modeling long-range trends or higher-order nonlinearities remains limited. In spatial interpolation modeling, the selection of correlation functions directly affects the accuracy of Kriging methods in characterizing spatial variability structures.

Traditional single correlation functions, while possessing distinct advantages, exhibit certain limitations when modeling complex spatial processes. Specifically, the Gaussian function tends to over-smooth local variations, the spherical function demonstrates inadequate performance in characterizing long-tail decay patterns, and the Matérn function, despite its enhanced flexibility, suffers from relatively low computational efficiency. To achieve more accurate characterization of multi-scale or heterogeneous spatial dependence features, a hybrid correlation function strategy is implemented through linear-weighted integration of different functional advantages. Furthermore, an Adaptive Hybrid Correlation Kriging (AHC-Kriging) is developed by employing heuristic optimization algorithms to determine optimal hybrid weights.

This study systematically evaluates Kriging modeling performance using individual correlation functions: Gaussian, spline, spherical, and Matérn5. Comprehensive comparative analyses are then conducted to assess the performance of the proposed AHC-Kriging approach incorporating linearly weighted combinations of these functions.

### 3.2. Improved Strategy of AHC-Kriging

This section elaborates on the adaptive weight hybrid correlation function strategy. The proposed method enhances model performance by dynamically adjusting the contribution weights of distinct correlation functions. Initially, a set of base correlation functions exhibiting complementary characteristics is selected. During the initialization phase, the system assigns randomized weighting coefficients to each correlation function, thereby constructing a preliminary hybrid correlation function model. The mathematical formulation is expressed as follows:

(17) $$\begin{array}{l} C_{H}\left[Z\left(x^{\left(i\right)}\right),Z\left(x^{\left(j\right)}\right)\right]=\sum_{n=1}^{N}\omega_{n}Corr_{n}\left[Z\left(x^{\left(i\right)}\right),Z\left(x^{\left(j\right)}\right)\right],\\ \sum_{n=1}^{N}\omega_{n}=1 \end{array}$$

where *C_H_*[*Z*(**x**^(*i*)^),*Z*(**x**^(*j*)^)] denotes the hybrid correlation function, *ω* represents the weighting coefficient, and *N* indicates the number of hybrid correlation functions. In this study, *N* = 2.

According to the fundamental principles of Kriging, the correlation matrix is given as follows:

(18) $$R=\left[\begin{array}{ccc} C_{H}\left[Z\left(x^{\left(1\right)}\right),Z\left(x^{\left(1\right)}\right)\right] & \ldots & C_{H}\left[Z\left(x^{\left(1\right)}\right),Z\left(x^{\left(n\right)}\right)\right]\\ \vdots & \ddots & \vdots \\ C_{H}\left[Z\left(x^{\left(n\right)}\right),Z\left(x^{\left(1\right)}\right)\right] & \ldots & C_{H}\left[Z\left(x^{\left(n\right)}\right),Z\left(x^{\left(n\right)}\right)\right] \end{array}\right]$$

Based on the theoretical framework of Kriging, the predictive expression of the surrogate model can be formulated as follows:

(19) $$\hat{y}\left(x_{new}\right)=f^{T}\left(x_{new}\right)\hat{\beta }+r^{T}R^{-1}\left(y-F\hat{\beta }\right),$$

where **f** denotes a vector of regression functions. **β** represents the vector of unknown coefficients and **r** constitutes a vector containing the correlations between the new prediction point **x***_new_* and existing sample points. The matrix **F** is defined as [**f**(**x**^(1)^), **f**(**x**^(2)^), …, **f**(**x**^(*n*)^)]*^T^*, while the response vector **y** contains the true output values [*y*(**x**^(1)^), *y*(**x**^(2)^), …, *y*(**x**^(*n*)^)]*^T^* for each of the *n* sample points. As evident from Equation (19), the weighting coefficient *ω* of the hybrid correlation function directly influences the predictive accuracy of the Kriging. Therefore, this study employs the root mean square error (RMSE) between the predicted outputs (*ŷ*) and the actual observed values (*yₜᵣᵤₑ*) of the sample set as the evaluation metric:

(20) $$\mathrm{RMSE}\left(\omega \right)=\sqrt{\frac{1}{N_{test}}{\sum_{i=1}^{N_{test}}\left[y_{true}\left(x\right)-\hat{y}\left(x,\omega \right)\right]}^{2}}$$

where *N_test_* is the number of test sample sets. Based on this metric, the system employs the Improved Subtraction-Average-Based Optimizer (ISABO) [45] to automatically adjust the weighting coefficients of each correlation function. Through iterative optimization, the algorithm progressively reduces the RMSE value until convergence is achieved.

(21) $$\omega =\arg\;\min\;\mathrm{RMSE}\left(\omega \right)$$

During the optimization process, the algorithm analyzes the contribution of each correlation function to the predictive outcome, subsequently increasing the weights of well-performing correlation functions while reducing those of underperforming ones. Unlike Kriging with fixed weights, the adaptive weight strategy proposed in this study can uncover dominant correlations, ensuring that the model can adapt to nonlinear problems. In contrast, fixed mixture models may allocate unreasonable weights in heterogeneous regions. Ultimately, the model converges to an optimal set of weight configurations, enabling the hybrid correlation function to most accurately characterize the feature distribution of the data. This strategy not only achieves the blending of correlation functions but also automatically adapts the model structure based on the specific data characteristics, significantly enhancing the adaptability and predictive accuracy of the Kriging model in complex scenarios.

### 3.3. Performance Analysis of AHC-Kriging

To validate the surrogate performance of AHC-Kriging, Kriging surrogate modeling is performed for the five benchmark functions in Table 5, which collectively represent diverse functional characteristics: the multimodal and highly nonlinear Michalewicz function exhibiting severe oscillations in higher dimensions; the one-dimensional nonstationary Gramacy & Lee function with pronounced discontinuities and steep gradients; the smooth quadratic Rotated Hyper-Ellipsoid function demonstrating rotational symmetry with a global minimum at the origin; the Three-Hump Camel function featuring three symmetrical local minima and moderate smoothness; and the essentially quadratic Zakharov function incorporating higher-order perturbations that enhance nonlinearity in high dimensions while maintaining global smoothness. This comprehensive evaluation spanning one-, two-, and three-dimensional functions rigorously assesses AHC-Kriging’s predictive capabilities across varying functional landscapes.

Table 6 and Table 7 systematically compare the predictive performance of Gaussian, spline, Matérn5, spherical, and hybrid methods including AHC-Kriging, Co-Kriging, and Stacked-GP on test functions F1 to F5. The results demonstrate that F1 and F2 functions exhibit strong robustness to different correlation function choices, with relatively small fluctuations in prediction accuracy metrics Q^2^ and RMSE. Specifically, the Q^2^ values of F1 remain stable between 0.82 and 0.87, while RMSE ranges from 0.075 to 0.089. Similarly, F2 shows good stability with Q^2^ values concentrated in the 0.87 to 0.89 range. This observation aligns with the inherent characteristics of these functions, which feature low complexity and high smoothness. In contrast, the predictive performance of F3 to F5 functions depends significantly on the choice of correlation functions. Particularly for F3, RMSE values vary substantially across different methods, ranging from 0.028 to 825.5, highlighting the sensitivity of complex functions to modeling approaches. Among all tested methods, hybrid approaches, especially the proposed AHC-Kriging, demonstrate superior overall performance. Taking F5 as an example, AHC-Kriging achieves a Q^2^ value of 0.97 while maintaining the lowest prediction variance, strongly validating the effectiveness of the adaptive weighting strategy in integrating the advantages of different correlation functions. This hybrid method not only enhances prediction stability for simple functions but, more importantly, significantly improves modeling accuracy for complex functions, providing a more reliable solution for complex function prediction in engineering applications.

**Figure 6:**
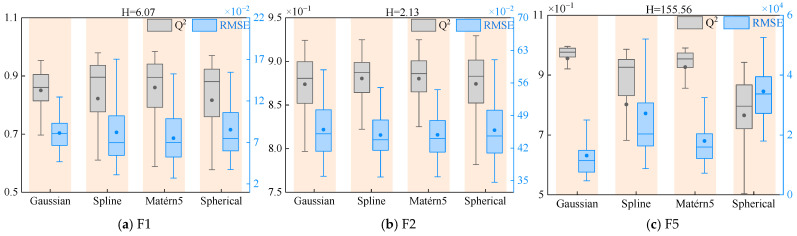
Kruskal–Wallis test results for F1, F2, and F5.

Based on the performance analysis results, this study employs the Kruskal–Wallis [49] nonparametric test to statistically analyze the Q^2^ and RMSE metrics of surrogate models under different correlation functions. The data in Table 5 and Table 6 show that F3 and F4 functions demonstrate excellent prediction accuracy across most surrogate models, with Q^2^ values consistently reaching 1, prompting subsequent analysis to focus on F1, F2, and F5 functions. Figure 6 visually presents the distribution characteristics of Q^2^ and RMSE for these three functions through boxplots, along with their corresponding H-statistics. With degrees of freedom set at 3 and significance level at 0.05, the critical value is determined as 7.815. The statistical test results reveal that the H-statistics for both F1 and F2 functions fall below this critical threshold, indicating no statistically significant differences in prediction performance among different correlation functions. In stark contrast, the H-statistic for the F5 function significantly exceeds the critical value, confirming that correlation function selection has a decisive impact on its prediction accuracy. These statistical inferences corroborate the preliminary performance analysis conclusions, enhancing the credibility of the research findings. The entire analytical process strictly adheres to the fundamental assumptions and applicable conditions of nonparametric statistical methods, ensuring the validity of the statistical conclusions.

### 3.4. Weighting Analysis Based on AHC-Kriging

This study conducts an uncertainty optimization for the laminated spherical-conical shell structure, with primary consideration given to material parameter uncertainties. The specific parameters and their value ranges are presented in Table 8, where the uncertainty level *δ* is set at 5%, indicating that the feasible interval for the uncertain parameters exhibits a 5% variation relative to their nominal values. To select appropriate hybrid correlation functions for Kriging model construction, this section focuses on the dynamic model of laminated spherical-conical shell structures described in Section 2, while incorporating the uncertain parameters from Table 8 for AHC-Kriging modeling and subsequent weight analysis.

This study systematically evaluates the prediction performance of Kriging models based on different correlation functions and hybrid methods for the displacement response of spherical-conical structures. Under clamped boundary conditions, an experimental design with training sample sizes incrementing by multiples of 7 from 14 to 175 was implemented, while maintaining a fixed prediction sample size of 1000 and ensuring result reliability through 80 repeated trials. As shown in Table 9 and Table 10, the experimental results demonstrate that model prediction accuracy significantly improves with increasing training sample size, with Gaussian, spline, and Matérn5 functions exhibiting the most outstanding performance. Their Q^2^ values approach 1 while maintaining extremely low RMSE levels, whereas the spherical function shows relatively inferior prediction capability. Notably, the Stacked-GP method demonstrates the poorest performance among all compared models, with significantly lower Q^2^ values and higher RMSE. After comprehensive consideration of computational efficiency and prediction accuracy, this study identifies 105 training samples as the optimal choice and selects Gaussian, spline, and Matérn5 functions as basis functions for the AHC-Kriging model, as this combination consistently demonstrates excellent and stable prediction capabilities across different sample sizes.

**Figure 7:**
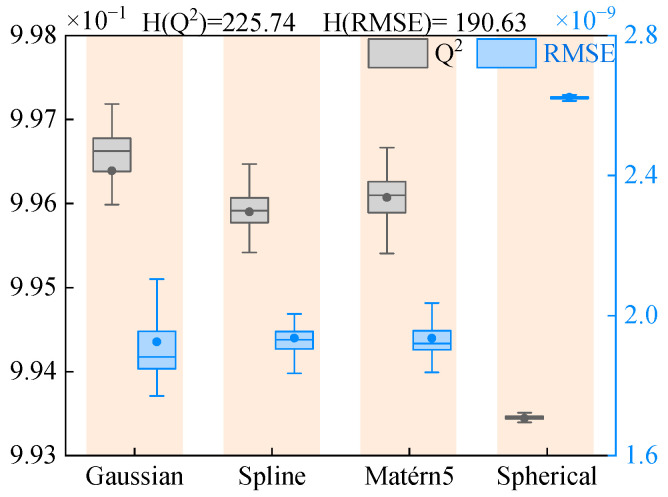
Results of the Kruskal–Wallis test for the displacement response of the laminated spherical-conical structure.

The boxplots of Q^2^ and RMSE distributions for the displacement response predictions of the spherical-conical structure, along with their corresponding H-statistic values, are presented in Figure 7. With the degrees of freedom set to 3 and the significance level at 0.05, the critical value is determined as 7.815. The analysis results demonstrate that the H-statistics for both Q^2^ and RMSE significantly exceed this critical threshold, providing conclusive statistical evidence that the selection of correlation functions critically determines the prediction accuracy of Kriging surrogate models. Based on this finding, subsequent research will primarily investigate the performance characteristics of surrogate models constructed with hybrid correlation functions.

Table 11 presents the hybrid types of three correlation functions along with the statistical metrics of prediction accuracy for the constructed AHC-Kriging surrogate model. It should be noted that “Weight” denotes the assigned weighting factor of the first correlation function in the hybrid correlation function listed in Table 11. As shown in the table, the prediction accuracy of the AHC-Kriging surrogate model exhibits improvement across all cases.

**Figure 8:**
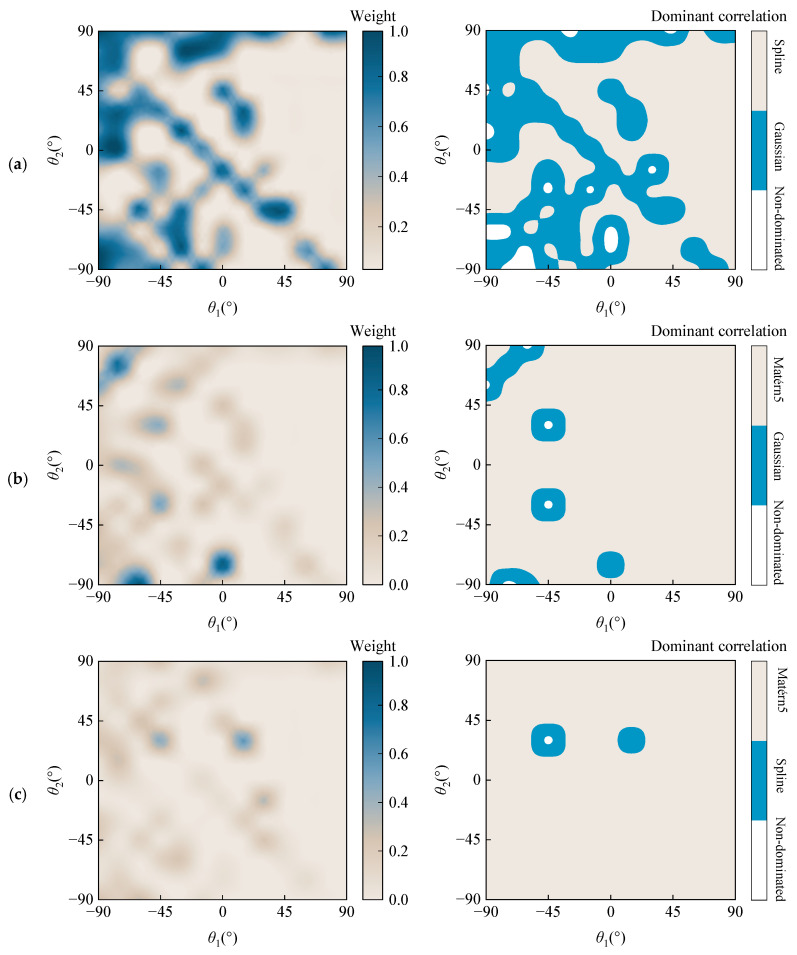
Distribution of weight of AHC-Kriging constructed by different hybrid correlation functions with ply angles: (**a**) Gaussian + Spline; (**b**) Gaussian + Matérn5; (**c**) Spline + Matérn5.

Figure 8 illustrates the weight distribution of the constructed AHC-Kriging surrogate models and the dominant correlation functions under varying ply angles. The selection of hybrid correlation functions aligns with the specifications in Table 11. As depicted in Figure 8a, the regions dominated by Gaussian and spline functions exhibit comparable areas. In contrast, Figure 8b,c demonstrate that the Matérn5 function assumes a predominant role. The left panels of Figure 8 present the weight distributions, revealing that the darker-colored regions remain consistent across all three cases. This observation indicates that when the ply angles fall within these specific ranges, the performance of the single correlation function is inferior to that of the hybrid correlation functions in constructing the Kriging surrogate model. These findings provide valuable insights for the subsequent development of Kriging models incorporating ply angle considerations.

## 4. Uncertainty Optimization of the Spherical-Conical Shell

### 4.1. Problem Statement for Optimization with Parametric Uncertainties

The uncertainty optimization problem concerning vibration characteristics in laminated spherical-conical shell structures can be mathematically formulated as follows:

(22) $$\begin{array}{l} min/\max:I\left(X,U\right)\\ find:X=\left[\theta_{1},\;\ldots ,\theta_{k}\right]\\ s.t:-90{}^{\circ}\leq \theta_{i}\leq 90{}^{\circ},\;i=1,\ldots ,k \end{array}$$

where *I* represents the vibration characteristics of laminated spherical-conical structures, **X** defines the design variable vector composed of ply angles in the composite laminate, and **U** encompasses the uncertain parameters. Each ply angle *θ_i_* is constrained within the range of [−90°, 90°], and *k* indicates the number of optimized ply angles.

According to Ref. [50], the interval analysis method is employed to study the uncertainty parameter interval **U**. The range of possible values of *I*(**X**,**U**) is an interval as follows:

(23) $$I(X,U)\in \left[I_{\min}\left(X\right),I_{\max}\left(X\right)\right]=\left[I^{c}\left(X\right)-I^{w}\left(X\right),I^{c}\left(X\right)+I^{w}\left(X\right)\right]$$

where *I^c^*(**X**) and *I^w^*(**X**) are specified by the following:

(24) $$I^{c}\left(X\right)=\frac{I_{\max}\left(X\right)+I_{\min}\left(X\right)}{2},I^{w}\left(X\right)=\frac{I_{\max}\left(X\right)-I_{\min}\left(X\right)}{2}$$

*I*_max_(**X**) and *I*_min_(**X**) are computationally addressed through an optimization algorithm:

(25) $$I_{\min}\left(X\right)=\underset{U}{\min}I\left(X,U\right),I_{\max}\left(X\right)=\underset{U}{\max}I\left(X,U\right)$$

Based on the above description, the optimization objective of this chapter can be expressed as follows:

(26) $$\begin{array}{l} min/max:\frac{\underset{U}{\max}I\left(X,U\right)+\underset{U}{\min}I\left(X,U\right)}{2}\\ \;\;\;\;\;\frac{\underset{U}{\max}I\left(X,U\right)-\underset{U}{\min}I\left(X,U\right)}{2}\\ find:\;X=\left[\theta_{1},\theta_{2}\right]\\ s.t:\;-90{}^{\circ}\leq \theta_{i}\leq 90{}^{\circ},\;i=1,2\\ \;\;U=\left[U_{1},\;\ldots ,U_{k}\right]\\ \;\;U_{j}=\left[U^{c}-0.5\delta U^{c},\;U^{c}+0.5\delta U^{c}\right],j=1,\ldots ,k \end{array}$$

**Figure 9:**
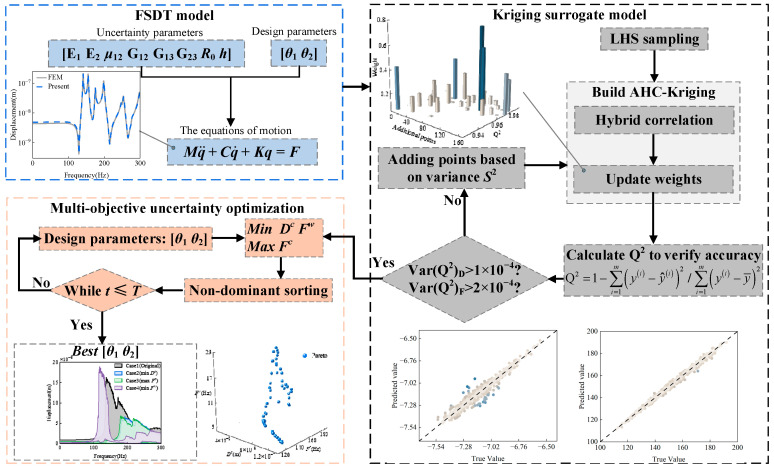
The whole optimization process.

The optimization framework, as depicted in Figure 9, commences with the development of a dynamic laminated spherical-conical shell model based on FSDT, which provides the fundamental basis for vibration displacement computation. Following this, an Adaptive Hybrid Correlation function-enhanced Kriging (AHC-Kriging) surrogate model is established to facilitate subsequent optimization, with its precision rigorously verified against predetermined criteria. The multi-objective optimization process ultimately yields the optimal Pareto frontier for *I^c^* and *I^w^*, from which the optimal design parameters (ply angles) are determined through comprehensive evaluation.

### 4.2. Improved Multi-Objective Salp Swarm Algorithm

In this study, the Multi-objective Salp Swarm Algorithm (MSSA) was selected as the optimizer over other conventional algorithms (e.g., NSGA-II and MOPSO) primarily due to its distinctive advantages in addressing optimization problems of composite shell structures. The MSSA, originally proposed by Mirjalili et al. [51], draws inspiration from the collective foraging behavior of salps in marine environments, where the population is organized into leader individuals that direct movement and follower individuals that establish chain formations through direct or indirect following. The algorithm emulates this unique chained foraging behavior to establish a leader–follower cooperative search mechanism. In this framework, leader individuals are responsible for guiding the search direction, while follower individuals achieve localized refinement through chain-based coordination. As demonstrated in previous successful applications of SSA reported in Ref. [52], this division-of-labor mechanism proves particularly suitable for addressing engineering problems with strong coupling characteristics, such as ply angle optimization in composite shell structures.

However, this leader-follower mechanism often leads to premature convergence and local optima trapping, while its fixed parameters limit robustness in handling high-dimensional or complex multi-objective problems, adversely affecting solution distribution and convergence accuracy. To address these limitations, this paper introduces an Improved Multi-objective Salp Swarm Algorithm (IMSSA) that incorporates the Sine Cosine Algorithm (SCA) [53] to enhance the leader’s global exploration capability and implements an adaptive nonlinear weighting strategy to dynamically adjust search step sizes—promoting exploration in early iterations while accelerating convergence in later stages, thereby mitigating leader dependency issues.

Under the initial assumption that there exists a food source designated as *F* within the search space serving as the population’s objective, the position update of leaders in MSSA is formulated as follows:

(27) $$x_{j}^{1}=\left\{\begin{array}{c} F_{j}+c_{1}\left[\left(ub_{j}-lb_{j}\right)c_{2}+lb_{j}\right],c_{3}\geq 0\\ F_{j}-c_{1}\left[\left(ub_{j}-lb_{j}\right)c_{2}+lb_{j}\right],c_{3}<0 \end{array}\right.$$

where $x_{j}^{1}$ denotes the position of the first salp (leader) in the *j*-th dimension, *F_j_* represents the food source’s location in the *j*-th dimension, *ub_j_* and *lb_j_* are the upper and lower bounds of the *j*-th dimension, respectively, and *c*_1_, *c*_2_, and *c*_3_ are random coefficients. After incorporating the SCA, the leader’s position is updated as follows:

(28) $$x_{j}^{1}=\left\{\begin{array}{c} F_{j}+r_{1}\cdot \sin\left(r_{2}\right)\cdot \left|r_{3}\cdot F_{j}-x_{j}^{1}\right|,r_{3}\leq 1\\ F_{j}+r_{1}\cdot \cos\left(r_{2}\right)\cdot \left|r_{3}\cdot F_{j}-x_{j}^{1}\right|,r_{3}>1 \end{array}\right.$$

where *r*_1_ = *a*·(2·*rand* − 1), *a* = 2 − *t*·(2/*T*), *t* is the current iteration, *T* is the maximum number of iterations, *r*_2_ denotes the angle between 0 and 2π, and *r*_3_ ∈ [0, 2] is a uniformly distributed random number that determines whether the sine or cosine mode is selected. Equation (28) achieves dynamic balance between exploration and exploitation through periodic oscillations of sine/cosine functions. During initial iterations, the larger amplitude parameter *r*_1_ combined with stochastic fluctuations of *r*_2_ facilitates global exploration to identify diverse Pareto solutions. As the optimization progresses, the linearly decaying *r*_1_ parameter reduces oscillation amplitudes, while the persistent stochasticity of *r*_2_ transitions the search towards local refinement.

In the MSSA, the position update of follower salps is formulated as follows:

(29) $$x_{j}^{i}=\frac{1}{2}\left(x_{j}^{i}+x_{j}^{i-1}\right)$$

When *i* ≥ 2, $x_{j}^{i}$ denotes the position of the *i*-th follower salp in the *j*-th dimension. The IMSSA introduces an adaptive nonlinear weighting factor to perturb the follower positions. Accordingly, the updated position of follower salps is expressed as follows:

(30) $$x_{j}^{i}=\frac{1}{2}\left(x_{j}^{i}+\omega \cdot x_{j}^{i-1}\right)$$

The expression for the adaptive nonlinear weights *ω* is as follows:

(31) $$\omega =\cos\left(\pi t\right)\cdot 5^{\left[\left(-\frac{4}{3}\right)\cdot \frac{t}{T}\right]}$$

The synergistic effect of SCA and *ω* effectively enhances both the distribution uniformity and convergence accuracy of the Pareto front in multi-objective optimization. This approach demonstrates particular efficacy when addressing complex problems characterized by high dimensionality, nonlinearity, or strongly conflicting objectives, while simultaneously maintaining population diversity and improving algorithmic robustness. Algorithm 1 presents the overall process (pseudo code) of the IMSSA.
**Algorithm 1.** The overall process of the IMSSA**Pseudo code**: IMSSASet problem parameters: population size (*N*), iterations (*T*), number of objectives, dimensions, and upper and lower bounds.
Randomly generate the initial salp population.
Evaluate the objective function.
**for** *t* = 1: *T* **do**
   **for**
*i* = 1: *N* **do**
      Evaluate the objective function.
      Update the position of food(*F*) in the current population.
   **end for**
   Update the repository.
   Chose the repository member in the least population area as food(*F*).
   **for**
*i* = 1: *N* **do**
      ***if*** *i* < *N*/2
     Update the position of the leader based on Equation (28).
    **else**
     Update the position of the followers based on Equations (30) and (31).
    **end *if***
    Correct for the salps based on the upper and lower bounds of variables.
  **end for**
**end for**
Return repository.

**Figure 10:**
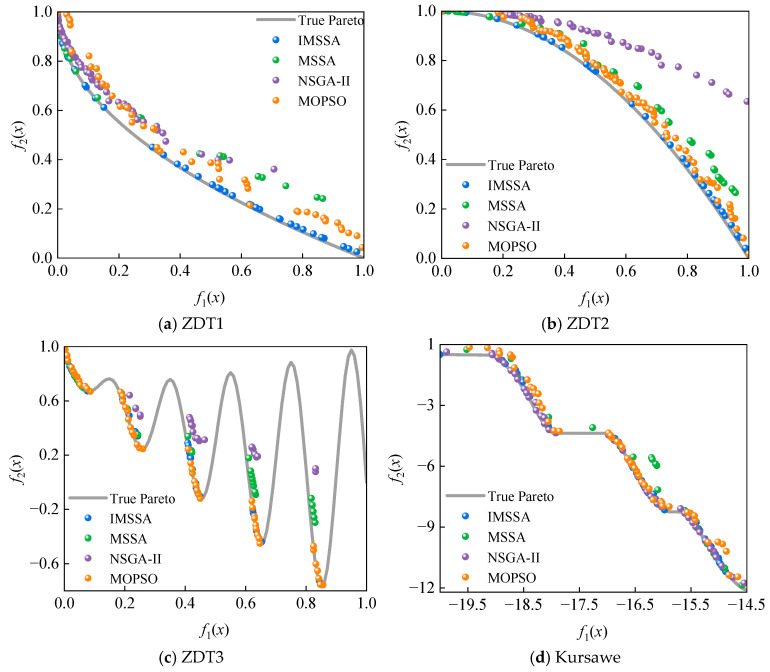
The performance of the four optimization algorithms on the test functions.

To evaluate the performance of the IMSSA, this section conducts comparative analyses with the MSSA and two classical optimization algorithms: the Non-dominated Sorting Genetic Algorithm II (NSGA-II) [54] and the Multi-Objective Particle Swarm Optimization (MOPSO) [55]. Table 12 presents the mathematical expressions and variable domains of four benchmark test functions [56]. Figure 10 illustrates the approximate Pareto fronts obtained by the four multi-objective optimization algorithms for classical multidimensional test functions, with comparative results against the true Pareto fronts. The scatter plot distribution reveals that the IMSSA achieves significantly higher congruence with the true Pareto front, demonstrating nearly identical geometric characteristics. This indicates IMSSA’s superior capability in effectively approximating the true Pareto front during solution searches. Furthermore, compared to the other three optimization algorithms, the IMSSA generates more uniformly distributed solutions along the approximate front. This property suggests IMSSA’s enhanced capacity for maintaining balanced trade-offs among competing objectives, thereby better preserving solution diversity and improving coverage of the objective space.

Ten optimization experiments are conducted on the test functions listed in Table 12 using IMSSA, MSSA, NSGA-II, and MOPSO. Table 13 presents the mean and variance of the generational distance (GD) metric. A comparison of the data reveals that for the Kursawe test function, the mean and variance of the results obtained by the IMSSA are slightly larger than those of NSGA-II. However, in all other cases, the IMSSA yields significantly smaller mean and variance values compared to the other three optimization algorithms. Overall, the IMSSA demonstrates superior convergence performance over the MSSA as well as the classical optimization algorithms NSGA-II and MOPSO. Table 14 provides the hypervolume (HV) indicator results. Comparative analysis shows that for the Kursawe test function, the IMSSA achieves a smaller mean value than MOPSO and a larger variance than NSGA-II. Nevertheless, in all other scenarios, the IMSSA exhibits significantly higher mean and lower variance values than the other three algorithms. This indicates that the IMSSA outperforms MSSA, NSGA-II, and MOPSO in terms of solution diversity. Subsequently, IMSSA will be applied to perform multi-objective uncertainty optimization on the spherical-conical shell structure.

### 4.3. Validation of AHC-Kriging for Uncertainty Optimization

According to the prior analysis, the material properties outlined in Section 3.4 will be identified as the uncertainty parameters. It should be noted that this also includes structural parameters: the uncertainty range for *R*_0_ is 0.45 to 0.55 m, while the uncertainty range for *h* is 0.035 to 0.045 m. These parameters, together with the design variables (ply angles), will serve as input variables for the AHC-Kriging surrogate models.

The study first develops Kriging models with four individual correlation functions (Gaussian, spline, Matérn5, and spherical) to predict maximum displacement and first frequency in laminated spherical-conical shells, incorporating both design and uncertain variables. This analysis helps select the optimal correlation function for constructing the AHC-Kriging surrogate models. The number of modeling samples is uniformly set to 220, and the number of prediction samples is uniformly set to 1000. By constructing the Kriging model 80 times, the mean and variance of the predicted Q^2^ and RMSE are calculated and presented in Table 15 and Table 16. The systematic analysis in Table 15 and Table 16 demonstrates that different correlation functions significantly influence the predictive performance of the Kriging surrogate model. For displacement response prediction, the Matérn5 and spherical correlation functions demonstrate optimal performance with a hybrid weighting coefficient of 0.0316, while the Gaussian and Matérn5 functions achieve superior accuracy in first-order structural frequency prediction with a hybrid weighting coefficient of 0.3636.

**Figure 11:**
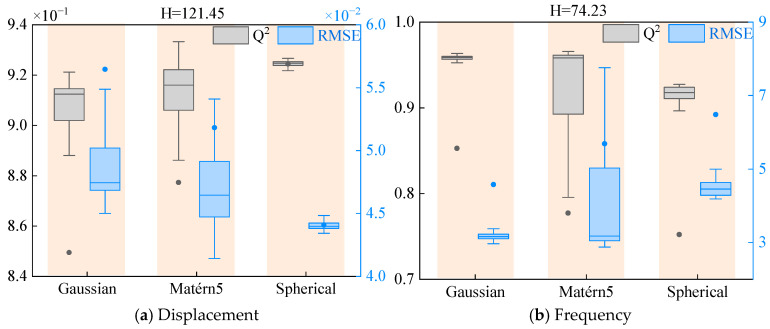
Results of the Kruskal–Wallis test for the displacement response and frequency of the laminated spherical-conical structure.

The distributions of Q^2^ and RMSE metrics for the AHC-Kriging surrogate model predictions of both displacement response and first-order frequency of the structure, along with their corresponding Kruskal–Wallis test statistic H-values are presented in Figure 11. Unlike previous analyses, the critical value is determined as 5.991 under test conditions with 2 degrees of freedom and a significance level of 0.05. The results demonstrate that the H-statistics for both Q^2^ and RMSE significantly exceed this critical threshold, providing robust statistical evidence that the choice of correlation functions exerts a decisive influence on the prediction accuracy of Kriging surrogate models. Based on the systematic performance evaluation, this study ultimately adopts a combination of Matérn5 and Spherical functions to construct the AHC-Kriging surrogate model for displacement response, while employing Gaussian and Matérn5 functions for the first-order frequency prediction model, thereby ensuring optimal prediction accuracy for different vibration characteristics.

**Figure 12:**
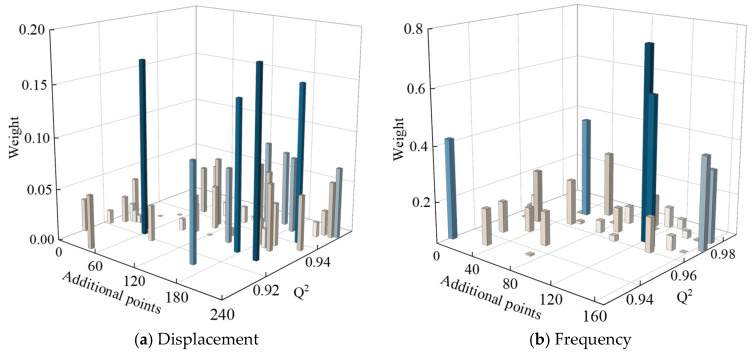
Performance of the Kriging surrogate models used for optimization as the number of additional sampling points increases.

**Figure 13:**
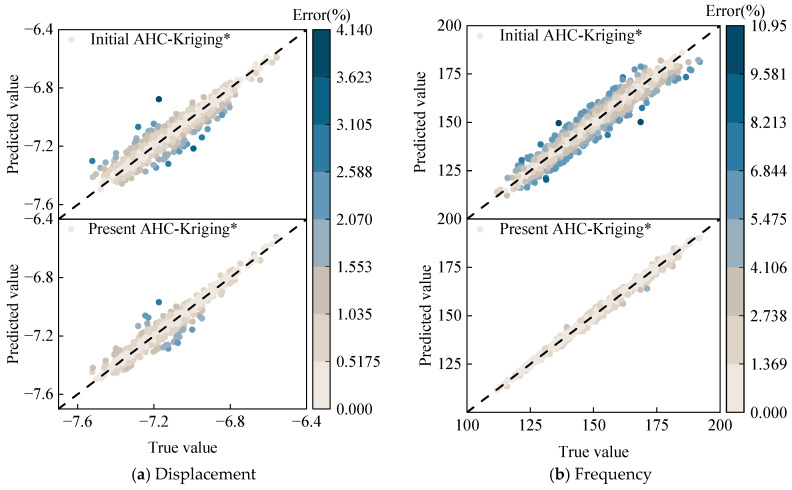
Accuracy comparison between real observations and model predictions for the AHC-Kriging surrogate model.

The subsequent procedure involves constructing AHC-Kriging surrogate models (denoted as AHC-Kriging*) for optimization purposes. The initial modeling sample size and prediction sample size remain consistent with the previously established settings. Through the adaptive sampling point update strategy, the predictive accuracy of this surrogate model is significantly enhanced. During the sequential sampling process, an early stopping mechanism is implemented, which terminates the updates when the variances of Q^2^ for both maximum displacement response and fundamental frequency fall below 1.0 × 10^−4^ and 2.0 × 10^−4^, respectively. This mechanism effectively prevents overfitting that may occur with excessive sampling points. The two thresholds are determined through pre-training experiments, where further reduction of Q^2^ variance beyond these values is observed to yield diminishing returns in model performance on the validation set. These thresholds achieve an optimal balance between computational efficiency and model robustness. The evolution of the fitting quality coefficient Q^2^ and weight parameters during the sampling point update process is illustrated in Figure 12, where data points are presented at 5-sample intervals for enhanced clarity. For maximum displacement response prediction (Figure 12a), the AHC-Kriging* model’s initial Q^2^ of 0.91 improved to 0.95 following the ISABO algorithm [45], with sampling point updates upon reaching 240 additional samples, and weight parameters consistently remaining below 0.2 and predominantly under 0.1 when excluding transient decreases during updates. Similarly, for fundamental frequency prediction (Figure 12b), the model’s initial Q^2^ of 0.93 increased to 0.98 after incorporating 160 additional samples, while weight parameters are distributed below 0.4. The comparative analysis in Figure 13 demonstrates the superior predictive performance of the updated surrogate model. As evidenced by the results, the maximum prediction error for displacement response decreases from 4.1% to 2.8%, while the maximum prediction error for fundamental frequency is reduced from 10.9% to 4.1%. These quantitative improvements substantiate the significant enhancement in model accuracy achieved through the adaptive sampling strategy.

### 4.4. Optimization Analysis

**Figure 14:**
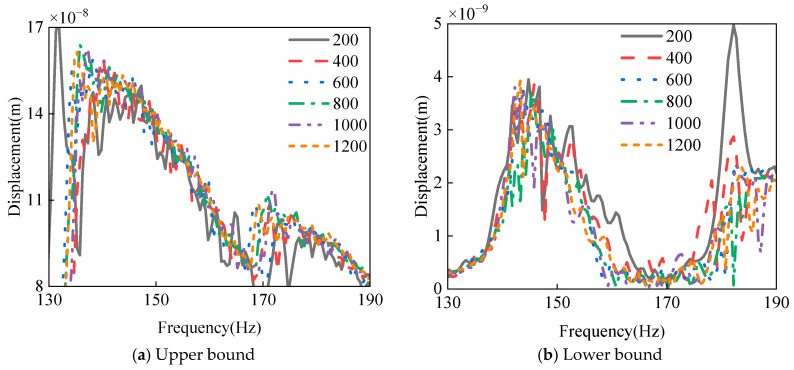
The upper and lower bound curves of displacement response uncertainty under different sample sizes.

This study conducts multi-objective uncertainty optimization based on the AHC-Kriging surrogate models established in Section 4.3 for the fundamental frequency and maximum displacement response of the laminated spherical-conical structure. The optimization objectives include the interval midpoint and radius of the uncertain maximum displacement response, as well as the negative value of the interval midpoint and the radius of the fundamental frequency. For the construction of uncertain response intervals, the Monte Carlo sampling method is employed, and rigorous interval convergence verification is performed. Taking the typical ply configuration [0/90°/90°/0] as an example, Table 17 systematically presents the Monte Carlo calculation results under different sample sizes. The analysis reveals that as the number of sampling points increases, the fluctuations of the four target parameters significantly decrease and eventually stabilize. Figure 14 displays the upper and lower bound curves of displacement response uncertainty under different sample sizes. As can be observed from the figure, when the sample size exceeds 800, the variation amplitude of the uncertainty curves becomes relatively small, with their trends remaining essentially consistent. Considering both computational efficiency and accuracy, this study ultimately selects 1000 sampling points for subsequent analysis, as this quantity ensures reliable results while maintaining reasonable computational costs.

**Figure 15:**
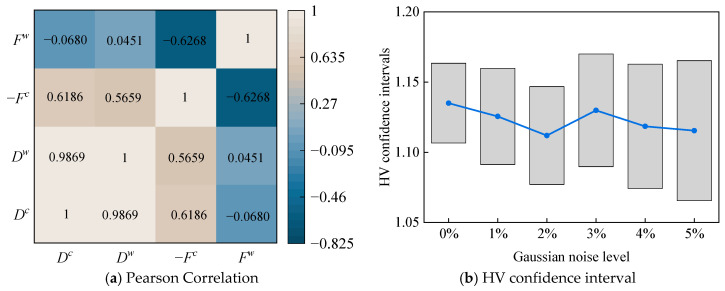
Optimization target selection and confidence detection of laminated spherical-conical structures.

To mitigate the potential impact of strong correlations between objective functions on the diversity of the Pareto front and prevent its degeneration into a limited solution set, this study employs the Pearson correlation coefficient [57] to quantitatively evaluate the interdependencies among the objective functions. As illustrated in Figure 15a, the interval midpoint *D^c^* and radius *D^w^* of the uncertain maximum displacement response exhibit a high degree of linear correlation, with a Pearson coefficient of 0.9869, while the correlations among the remaining objective functions remain relatively weak. Based on this analysis, the final optimization model retains only three independent objective functions: the interval midpoint of the maximum displacement response, the negative value of the interval midpoint of the fundamental frequency, and its corresponding interval radius. During the optimization process, the population size and maximum iteration count of the external IMSSA algorithm are both set to 50 to ensure sufficient global search capability. The confidence bands around the Pareto front and the sensitivity of trade-offs to model noise were investigated. After normalization, the multidimensional frontier distribution is generated via Monte Carlo sampling, and the confidence intervals of the hypervolume (HV) are computed based on all sampled solutions. The coordinates of the reference point are (1.1, 1.1, 1.1). Gaussian noise with varying standard deviations is introduced, and the distribution of the 95% HV confidence intervals is presented in Figure 15b. The results show that the confidence intervals remain narrow, indicating high stability of the Pareto front. Moreover, as the Gaussian noise level increases, the confidence intervals widen, but only marginally, demonstrating the considerable robustness of the Pareto front.

The results of multi-objective uncertainty optimization are presented in Table 18, along with a comparative analysis against the original-displacement response (Case 1). Among them, Case 2 represents the scenario with the smallest *D^c^* in the optimization results, Case 3 corresponds to the optimized outcome with the largest *F^c^*, and Case 4 denotes the optimization scheme with the smallest *F^w^*. The data reveal that the maximum reductions in *D^c^* and *D^w^* reach 58.58% and 60.60%, respectively, while *F^c^* achieves a maximum increase of 35.25%, and *F^w^* exhibits a maximum reduction of 61.58%. These results fully demonstrate that the proposed uncertainty optimization method in this study can effectively suppress the vibration response of the laminated spherical conical shell structure and significantly enhance its dynamic performance.

**Figure 16:**
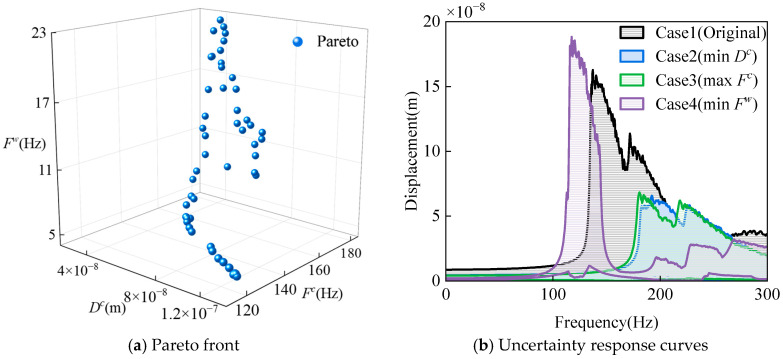
Visualization of uncertainty optimization results for laminated spherical-conical shell structure.

To further analyze the effectiveness of uncertainty optimization, Figure 16 illustrates the Pareto front and the uncertainty response curves for Cases 1–4. Figure 16a indicates that as the median of the displacement response interval increases, both the median and radius of the frequency response interval exhibit a decreasing trend. Figure 16b specifically presents the optimization results from Table 18, where the uncertainty response curves demonstrate an envelope-like characteristic. Compared with the original response interval, the response intervals for Cases 2 and 3 are significantly reduced, and the overall envelope curve shifts toward higher frequencies, indicating a decrease in the displacement response interval radius and an increase in the frequency interval radius. In contrast, the response peak of Case 4 rises slightly, with the envelope curve shifting toward lower frequencies and narrowing in width, reflecting a reduction in the frequency interval radius.

## 5. Conclusions

This study proposes a Kriging surrogate model based on adaptive hybrid correlation functions for multi-objective uncertainty optimization of displacement responses in laminated spherical-conical shell structures, along with an improved Multi-objective Salp Swarm Algorithm. The key findings are summarized as follows: (1) The hybrid correlation function effectively integrates the advantages of different correlation functions, enhancing the prediction accuracy of the Kriging surrogate model. (2) For the displacement response of laminated spherical-conical shell structures, the weights of the constructed AHC-Kriging vary with the ply angles. (3) The improved Multi-objective Salp Swarm Algorithm significantly enhances both the convergence and diversity of the Pareto front. (4) The proposed uncertainty-based multi-objective optimization methodology effectively reduces both the midpoint value and radius of the displacement response uncertainty interval, while simultaneously decreasing the radius and increasing the midpoint value of the fundamental frequency uncertainty interval. The results demonstrate maximum reductions of 58.58% for *D^c^* and 60.60% for *D^w^*, along with a maximum increase of 35.25% for *F^c^* and maximum reduction of 61.58% for *F^w^*. These significant improvements substantially enhance the structure’s vibration resistance capability. However, this study has certain limitations. On one hand, only clamped boundary conditions are considered for the laminated spherical-conical shell structure, whereas elastic connections between structures are common in practical engineering scenarios, making the exclusive consideration of clamped boundaries insufficient. On the other hand, only material parameter uncertainties are accounted for, which is not comprehensive. Future research will incorporate elastic connections and more extensive uncertainty parameters to address these gaps.

## Figures and Tables

**Table 1:** The stiffness parameters defining the elastic constraints in generalized boundary conditions.

Boundary Set	*k_u_*	*k_v_*	*k_w_*	*k_α_*	*k_β_*
C (Clamped)	10^14^	10^14^	10^14^	10^14^	10^14^
F (Free)	0	0	0	0	0

**Table 2:** Natural frequencies of laminated spherical-cylindrical shell structures with varying axial displacement function truncation numbers.

Mode	Axial Displacement Function Truncation Numbers							Diff. (%)
	M = 8	M = 10	M = 12	M = 14	M = 16	M = 18	Ref. [44]	
1	49.41	49.37	49.33	49.32	49.32	49.32	49.35	0.0608
2	137.43	137.31	137.20	137.17	137.17	137.17	137.2	0.0219
3	262.02	261.81	261.61	261.55	261.54	261.54	261.6	0.0229
4	364.52	364.35	364.19	364.13	364.12	364.12	363.5	0.1706
5	408.23	408.17	408.11	408.09	408.08	408.08	407.9	0.0441

**Table 3:** The vibrational frequencies (Hz) for laminated spherical-conical shells under varying lamination schemes and boundary conditions.

B.C	Mode	[0°/90°/90°/0°]			[−45°/45°/45°/−45°]		
		Present	FEM	Diff. (%)	Present	FEM	Diff. (%)
C	1	127.99	127.81	0.1406	167.12	166.71	0.2453
	3	141.05	141.00	0.0394	180.06	180.02	0.0222
	5	155.44	155.10	0.2187	190.46	190.15	0.1628
	7	175.05	175.06	0.0057	203.39	202.79	0.2950
	9	199.08	198.54	0.2712	253.90	253.16	0.2915
F	7	17.52	17.38	0.7991	22.80	22.23	2.5000
	9	44.10	43.96	0.3175	51.32	50.86	0.8963
	11	75.07	74.86	0.2797	85.84	85.32	0.6058
	13	113.10	112.77	0.2918	128.20	127.61	0.4602
	15	135.84	135.52	0.2356	146.03	145.15	0.6026

**Table 4:** Commonly used correlation functions and their expressions.

Correlation Function	Expression
Gaussian	$\exp\left[-\sum_{h=1}^{k}\theta_{h}{\left(x_{h}^{\left(i\right)}-x_{h}^{\left(j\right)}\right)}^{2}\right]$
Spline	$\left\{\begin{array}{l} 1-15{\left(\sum_{h=1}^{k}\theta_{h}\left|x_{h}^{\left(i\right)}-x_{h}^{\left(j\right)}\right|\right)}^{2}+30{\left(\sum_{h=1}^{k}\theta_{h}\left|x_{h}^{\left(i\right)}-x_{h}^{\left(j\right)}\right|\right)}^{3},\;0\leq \sum_{h=1}^{k}\theta_{h}\left|x_{h}^{\left(i\right)}-x_{h}^{\left(j\right)}\right|\leq 0.2\\ 1.25{\left[1-\left(\sum_{h=1}^{k}\theta_{h}\left|x_{h}^{\left(i\right)}-x_{h}^{\left(j\right)}\right|\right)\right]}^{3},\;0.2<\sum_{h=1}^{k}\theta_{h}\left|x_{h}^{\left(i\right)}-x_{h}^{\left(j\right)}\right|<1\\ 0,\;\sum_{h=1}^{k}\theta_{h}\left|x_{h}^{\left(i\right)}-x_{h}^{\left(j\right)}\right|\geq 1 \end{array}\right.$
Matérn (*v* = 5/2)	$\left(1+\sqrt{5}l+\frac{5l^{2}}{3}\right)\cdot \exp\left(-\sqrt{5l}\right),l=\sqrt{\sum_{h=1}^{k}\theta_{h}{\left(x_{h}^{\left(i\right)}-x_{h}^{\left(j\right)}\right)}^{2}}$
Spherical	$1-1.5\xi_{j}+0.5\xi_{j}^{3},\xi =\min\left\{1,\sum_{h=1}^{k}\theta_{h}{\left(x_{h}^{\left(i\right)}-x_{h}^{\left(j\right)}\right)}^{2}\right\}$

**Table 5:** Kriging surrogate modeling test functions.

Function	Expression	Dimensions	Range
Michalewicz Function (F1)	$f\left(x\right)=-\sum_{i=1}^{1}\sin\left(x_{i}\right)\sin^{20}\left(\frac{ix_{i}^{2}}{\pi }\right)$	1	[0, π]
Gramacy & Lee Function (F2)	$f\left(x\right)=\frac{\sin\left(10\pi x\right)}{2x}+{\left(x-1\right)}^{4}$	1	[0.5, 2.5]
Rotated Hyper-Ellipsoid Function (F3)	$f\left(x\right)=\sum_{i=1}^{2}\sum_{j=1}^{i}x_{j}^{2}$	2	[−65.536, 65.536]
Three-Hump Camel Function (F4)	$f\left(x\right)=2x_{1}^{2}-1.05x_{1}^{4}+\frac{x_{1}^{6}}{6}+x^{1}x^{2}+x_{2}^{2}$	2	[−5, 5]
Zakharov Function (F5)	$f\left(x\right)=\sum_{i=1}^{3}x_{i}^{2}+{\left(\sum_{i=1}^{3}0.5ix_{i}\right)}^{2}+{\left(\sum_{i=1}^{3}0.5ix_{i}\right)}^{4}$	3	[−5, 10]

**Table 6:** The mean values and variances of the fitting quality coefficient Q^2^ obtained from 80 Kriging predictions.

Function	Correlation Function														
	Gaussian		Spline		Matérn5		Spherical		AHC-Kriging			Co-Kriging		Stacked-GP	
	Average	Variance	Average	Variance	Average	Variance	Average	Variance	Average	Variance	Weight	Average	Variance	Average	Variance
F1	0.85	5.4 × 10^−3^	0.82	3.6 × 10^−2^	0.86	1.2 × 10^−2^	0.82	2.8 × 10^−2^	0.87	5.6 × 10^−3^	0.875	0.82	1.0 × 10^−2^	0.84	2.4 × 10^−2^
F2	0.87	1.2 × 10^−3^	0.88	9.1 × 10^−4^	0.88	9.1 × 10^−4^	0.87	1.2 × 10^−3^	0.89	6.9 × 10^−4^	0.026	0.88	7.3 × 10^−4^	0.93	5.2 × 10^−3^
F3	1	2.4 × 10^−20^	0.98	2.5 × 10^−6^	1	3.9 × 10^−17^	0.92	5.7 × 10^−4^	1	1.7 × 10^−10^	0.116	1	7.5 × 10^−14^	1	5.9 × 10^−8^
F4	1	4.7 × 10^−6^	0.99	8.4 × 10^−6^	1	2.0 × 10^−6^	0.95	1.2 × 10^−3^	1	4.8 × 10^−7^	0.416	0.98	4.5 × 10^−4^	0.93	4.9 × 10^−2^
F5	0.96	8.3 × 10^−3^	0.80	1.1 × 10^−1^	0.93	1.6 × 10^−2^	0.77	2.7 × 10^−2^	0.97	1.0 × 10^−3^	0.473	0.95	4.9 × 10^−3^	0.95	2.5 × 10^−3^

**Table 7:** The mean values and variances of the RMSE obtained from 80 Kriging predictions.

Function	Correlation Function														
	Gaussian		Spline		Matérn5		Spherical		AHC-Kriging			Co-Kriging		Stacked-GP	
	Average	Variance	Average	Variance	Average	Variance	Average	Variance	Average	Variance	Weight	Average	Variance	Average	Variance
F1	0.081	3.8 × 10^−4^	0.082	1.6 × 10^−3^	0.075	9.0 × 10^−4^	0.085	1.3 × 10^−3^	0.077	4.4 × 10^−4^	0.875	0.089	7.3 × 10^−4^	0.078	1.5 × 10^−3^
F2	0.460	3.6 × 10^−3^	0.448	3.0 × 10^−3^	0.449	3.0 × 10^−3^	0.459	4.0 × 10^−3^	0.443	2.4 × 10^−3^	0.026	0.457	2.5 × 10^−3^	0.389	2.5 × 10^−2^
F3	0.028	2.8 × 10^−4^	120.1	2.5 × 10^3^	0.282	7.9 × 10^−3^	825.5	1.4 × 10^4^	0.322	3.5 × 10^−3^	0.116	0.487	6.2 × 10^−1^	0.840	6.2 × 10^−1^
F4	25.93	71.41	33.28	79.93	25.34	37.56	87.82	1.3 × 10^4^	24.62	9.974	0.416	57.08	938.9	54.50	1.4 × 10^4^
F5	1.3 × 10^4^	7.8 × 10^7^	2.7 × 10^4^	3.7 × 10^8^	1.8 × 10^4^	8.9 × 10^7^	3.5 × 10^4^	1.2 × 10^8^	1.3 × 10^4^	3.0 × 10^7^	0.473	1.3 × 10^4^	1.1 × 10^6^	1.4 × 10^4^	4.5 × 10^7^

**Table 8:** Key parameters required for establishing the Kriging surrogate model.

Variables	Range
*E*_1_/GPa	[142.5, 157.5]
*E*_2_/GPa	[9.5, 10.5]
*μ*_12_	[0.2375, 0.2625]
*G*_12_/GPa	[5.7, 6.3]
*G*_13_/GPa	[5.7, 6.3]
*G*_23_/GPa	[4.75, 5.25]
*ρ*/kg·m^−3^	[1377.5, 1522.5]

**Table 9:** The average of Q^2^ for predictions made by Kriging constructed with individual correlation functions and other surrogates.

Surrogate	14	35	70	105	140	175
	Average	Average	Average	Average	Average	Average
Kriging–Gaussian	0.969	0.989	0.993	0.996	0.997	0.998
Kriging–spline	0.935	0.982	0.996	0.996	0.997	0.998
Kriging–Matérn5	0.972	0.989	0.996	0.996	0.997	0.998
Kriging–spherical	0.958	0.987	0.992	0.993	0.994	0.996
Co-Kriging	0.931	0.989	0.996	0.996	0.997	0.997
Stacked-GP	0.766	0.792	0.816	0.831	0.851	0.875

**Table 10:** The average of RMSE for predictions made by Kriging constructed with individual correlation functions and other surrogates.

Surrogate	14	35	70	105	140	175
	Average	Average	Average	Average	Average	Average
Kriging–Gaussian	4.7859 × 10^−9^	2.9287 × 10^−9^	2.2708 × 10^−9^	1.9247 × 10^−9^	1.7091 × 10^−9^	1.3201 × 10^−9^
Kriging–spline	6.1931 × 10^−9^	3.3125 × 10^−9^	1.9922 × 10^−9^	1.9353 × 10^−9^	1.5768 × 10^−9^	1.3777 × 10^−9^
Kriging-Matérn5	4.5482 × 10^−9^	2.9493 × 10^−9^	2.0432 × 10^−9^	1.9351 × 10^−9^	1.5244 × 10^−9^	1.3934 × 10^−9^
Kriging–spherical	5.0981 × 10^−9^	3.4330 × 10^−9^	2.8111 × 10^−9^	2.6229 × 10^−9^	2.2172 × 10^−9^	2.0798 × 10^−9^
Co-Kriging	5.7623 × 10^−9^	2.9657 × 10^−9^	2.0749 × 10^−9^	1.9890 × 10^−9^	1.6152 × 10^−9^	1.4120 × 10^−9^
Stacked-GP	9.4869 × 10^−9^	9.2354 × 10^−9^	9.1748 × 10^−9^	8.3178 × 10^−9^	8.1108 × 10^−9^	7.6898 × 10^−9^

**Table 11:** The statistical measures of Q^2^ and RMSE for predictions made by AHC-Kriging constructed with hybrid correlation functions.

Hybrid Correlation Function	Weight	Average of Q^2^	Average of RSME	Variance of Q^2^	Variance of RSME
Gaussian + Spline	0.9299	0.997	1.8620 × 10^−9^	8.8759 × 10^−7^	3.3626 × 10^−20^
Gaussian + Matérn5	0.2720	0.997	1.7844 × 10^−9^	1.7453 × 10^−7^	9.1235 × 10^−21^
Spline + Matérn5	0.1868	0.998	1.6975 × 10^−9^	2.4369 × 10^−8^	1.8450 × 10^−21^

**Table 12:** Test functions and variable ranges.

Test Functions	Expression	Dimensions	Range
ZDT1	$\begin{array}{l} F=\left(f_{1}\left(x\right),f_{2}\left(x\right)\right)\\ f_{1}\left(x_{1}\right)=x_{1},f_{2}\left(x\right)=1-\sqrt{\left(f_{1}\left(x\right)/g\left(x\right)\right)}\\ g\left(x\right)=1+9\sum_{i-2}^{D}x_{i}/\left(D-1\right) \end{array}$	30	[0, 1]
ZDT2	$\begin{array}{l} F=\left(f_{1}\left(x\right),f_{2}\left(x\right)\right)\\ f_{1}\left(x_{1}\right)=x_{1},f_{2}\left(x\right)=1-{\left(f_{1}\left(x\right)/g\left(x\right)\right)}^{2}\\ g\left(x\right)=1+9\sum_{i-2}^{D}x_{i}/\left(D-1\right) \end{array}$	30	[0, 1]
ZDT3	$\begin{array}{l} F=\left(f_{1}\left(x\right),f_{2}\left(x\right)\right)\\ f_{1}\left(x_{1}\right)=x_{1}\\ f_{2}\left(x\right)=1-\sqrt{\left(f_{1}\left(x\right)/g\left(x\right)\right)}-\left(f_{1}\left(x\right)/g\left(x\right)\right)\sin\left(10\pi f_{1}\left(x\right)\right)\\ g\left(x\right)=1+9\sum_{i-2}^{D}x_{i}/\left(D-1\right) \end{array}$	30	[0, 1]
Kursawe	$\begin{array}{l} F=\left(f_{1}\left(x\right),f_{2}\left(x\right)\right)\\ f_{1}\left(x\right)=\sum_{i=1}^{D-1}\left(-10e^{\left(-0.2\right)\times \sqrt{x_{i}^{2}-x_{i+1}^{2}}}\right),f_{2}\left(x\right)=\sum_{i=1}^{D}\left({\left|x_{i}\right|}^{0.8}+5\sin{\left(x_{i}\right)}^{3}\right) \end{array}$	3	[−5, 5]

**Table 13:** Mean and variance of generational distance (GD) metrics for different optimization algorithms.

Test Functions	Indicators	IMSSA	MSSA	NSGA-II	MOPSO
ZDT1	Average	0.0020	0.0095	0.0356	0.0354
	Std.	7.727 × 10^−7^	2.284 × 10^−6^	2.856 × 10^−5^	2.739 × 10^−6^
ZDT2	Average	0.0032	0.0135	0.0391	0.0326
	Std.	7.676 × 10^−6^	8.294 × 10^−6^	2.886 × 10^−4^	8.191 × 10^−6^
ZDT3	Average	0.0053	0.0107	0.0316	0.0131
	Std.	1.648 × 10^−5^	4.546 × 10^−5^	2.403 × 10^−5^	7.329 × 10^−6^
Kursawe	Average	0.0106	0.0316	0.0070	0.0392
	Std.	1.733 × 10^−5^	2.347 × 10^−4^	2.262 × 10^−6^	6.863 × 10^−4^

**Table 14:** Mean and variance of hypervolume (HV) metrics for different optimization algorithms.

Test Functions	Indicators	IMSSA	MSSA	NSGA-II	MOPSO
ZDT1	Average	0.6767	0.5368	0.3818	0.3652
	Std.	9.819 × 10^−6^	2.341 × 10^−4^	0.0016	4.923 × 10^−5^
ZDT2	Average	0.3918	0.1905	0.1334	0.1073
	Std.	1.189 × 10^−4^	2.978 × 10^−4^	1.747 × 10^−4^	1.450 × 10^−6^
ZDT3	Average	0.6116	0.5184	0.3922	0.6012
	Std.	7.648 × 10^−4^	0.0049	0.0021	9.840 × 10^−4^
Kursawe	Average	0.5085	0.4986	0.5010	0.5899
	Std.	0.0027	0.0036	2.857 × 10^−5^	0.0063

**Table 15:** The statistical measures of Q^2^ and RMSE for maximum displacement predictions made by Kriging constructed with individual correlation functions and other surrogates.

Surrogate	Average of Q^2^	Average of RMSE	Variance of Q^2^	Variance of RMSE
Kriging–Gaussian	0.8495	5.6457 × 10^−2^	4.0534 × 10^−2^	6.9082 × 10^−4^
Kriging–spline	0.5568	9.2001 × 10^−2^	1.9463 × 10^−1^	2.9661 × 10^−3^
Kriging–Matérn5	0.8873	5.1816 × 10^−2^	2.7555 × 10^−2^	4.7375 × 10^−4^
Kriging–spherical	0.9244	4.4087 × 10^−2^	2.0621 × 10^−6^	1.7236 × 10^−7^
AHC-Kriging	0.9255	4.3748 × 10^−2^	1.5420 × 10^−5^	1.3326 × 10^−6^
Co-Kriging	0.8664	1.2103 × 10^−1^	1.2738 × 10^−3^	2.7781 × 10^−3^
Stacked-GP	0.8266	2.1796 × 10^−1^	3.5762 × 10^−2^	4.2334 × 10^−2^

**Table 16:** The statistical measures of Q^2^ and RMSE for fundamental frequency predictions made by Kriging constructed with individual correlation functions and other surrogates.

Surrogate	Average of Q^2^	Average of RMSE	Variance of Q^2^	Variance of RMSE
Kriging–Gaussian	0.8528	4.5773	8.4366 × 10^−2^	14.774
Kriging–spline	0.3468	11.172	1.9722 × 10^−1^	33.313
Kriging–Matérn5	0.7771	5.6911	1.1666 × 10^−1^	21.694
Kriging–spherical	0.7522	6.4808	1.2180 × 10^−1^	18.046
AHC-Kriging	0.9238	4.0970	5.0279 × 10^−3^	1.6290
Co-Kriging	0.8198	5.6620	8.7001 × 10^−2^	12.716
Stacked-GP	0.8263	5.3319	7.2392 × 10^−2^	14.998

**Table 17:** Optimization results of the laminated spherical-conical shell structure.

Obj.	200	400	600	800	1000	1200
*D^c^*	8.9219 × 10^−8^	8.1390 × 10^−8^	8.1565 × 10^−8^	8.3844 × 10^−8^	8.3287 × 10^−8^	8.3194 × 10^−8^
*D^w^*	8.4250 × 10^−8^	7.7539 × 10^−8^	7.7828 × 10^−8^	7.9828 × 10^−8^	7.9486 × 10^−8^	7.9266 × 10^−8^
*F^c^*	132.3364	133.3849	132.5577	132.3604	131.8038	131.8683
*F^w^*	13.0238	15.3907	15.5161	16.0973	15.9519	15.8611

**Table 18:** Optimization results of the laminated spherical-conical shell structure.

Case	*θ*_1_/°	*θ*_2_/°	*D^c^* (×10^−8^ m)	Diff.(%)	*D^w^* (×10^−8^ m)	Diff.(%)	*F^c^*(Hz)	Diff. (%)	*F^w^* (Hz)	Diff. (%)
1 (Original)	0	90	8.3287	——	7.9486	——	131.8038	——	15.9519	——
2 (min *D^c^*)	51.45	−34.13	3.4501	58.58	3.1316	60.60	175.4543	33.1178	21.7264	36.1994
3 (max *F^c^*)	50.48	−18.03	3.5248	57.68	3.3083	58.38	178.2618	35.2478	19.8576	24.4842
4 (min *F^w^*)	−90	−90	10.0058	20.14	8.8292	11.08	119.1401	9.6080	6.1280	61.5845

## Data Availability

The original contributions presented in this study are included in the article. Further inquiries can be directed to the corresponding author.
